# A Macroecological Analysis of SERA Derived Forest Heights and Implications for Forest Volume Remote Sensing

**DOI:** 10.1371/journal.pone.0033927

**Published:** 2012-03-23

**Authors:** Matthew Brolly, Iain H. Woodhouse, Karl J. Niklas, Sean T. Hammond

**Affiliations:** 1 Department of Geographical Sciences, University of Maryland, College Park, Maryland, United States of America; 2 School of GeoSciences, The University of Edinburgh, Edinburgh, United Kingdom; 3 Department of Plant Biology, Cornell University, Ithaca, New York, United States of America; 4 Department of Ecology and Evolutionary Biology, University of Arizona, Tucson, Arizona, United States of America; University of Bristol, United Kingdom

## Abstract

Individual trees have been shown to exhibit strong relationships between DBH, height and volume. Often such studies are cited as justification for forest volume or standing biomass estimation through remote sensing. With resolution of common satellite remote sensing systems generally too low to resolve individuals, and a need for larger coverage, these systems rely on descriptive heights, which account for tree collections in forests. For remote sensing and allometric applications, this height is not entirely understood in terms of its location. Here, a forest growth model (SERA) analyzes forest canopy height relationships with forest wood volume. Maximum height, mean, H_100_, and Lorey's height are examined for variability under plant number density, resource and species. Our findings, shown to be allometrically consistent with empirical measurements for forested communities world-wide, are analyzed for implications to forest remote sensing techniques such as LiDAR and RADAR. Traditional forestry measures of maximum height, and to a lesser extent H_100_ and Lorey's, exhibit little consistent correlation with forest volume across modeled conditions. The implication is that using forest height to infer volume or biomass from remote sensing requires species and community behavioral information to infer accurate estimates using height alone. SERA predicts mean height to provide the most consistent relationship with volume of the height classifications studied and overall across forest variations. This prediction agrees with empirical data collected from conifer and angiosperm forests with plant densities ranging between 10^2^–10^6^ plants/hectare and heights 6–49 m. Height classifications investigated are potentially linked to radar scattering centers with implications for allometry. These findings may be used to advance forest biomass estimation accuracy through remote sensing. Furthermore, Lorey's height with its specific relationship to remote sensing physics is recommended as a more universal indicator of volume when using remote sensing than achieved using either maximum height or H_100_.

## Introduction

Accurate global forest inventory and above ground biomass estimates remain an uncertain element in our understanding of the global carbon cycle [1], [2]. Remote sensing by current and future techniques using SAR and LiDAR are expected to play an increasing role in reducing such uncertainties; alone, and in synergy [3]. Both of these techniques suffer from inaccuracies associated with their estimation of biomass. For SAR there are empirical results showing that relationships exist between the intensity of backscatter and the biomass of a forest so that an accurate estimate of biomass can be determined directly, but this technique is hampered by the existence of a saturation effect [4], [5] seen both in empirical [4] and theoretical studies [6], and through a lack of consistency across different forest types. The source of the saturation effect and the information that can be extracted at volumes above this saturation biomass are a topic of debate [5], [7], [8], [9]. A significant problem is that approximately 81% of the world's forests contain biomass beyond the saturation level currently associated with P-Band SAR [5] – the frequency of choice for the proposed European Space Agency mission, BIOMASS [10].

For SAR height, values can be inferred from polarimetric-interferometric radar [11]. The estimation accuracy, with respect to forest height, has been shown to be in the order of 10–15% for particular studies [12] but still requires the use of allometric equations to convert to biomass. For LiDAR the relationship between the LiDAR return and the height of the forest is more direct, with uncertainties associated largely with footprint size. For both large footprint (>10 m) LiDAR and SAR, the direct relation to “canopy height” as measured in the field is not well-defined, and different methods of calculating a mean, or representative height are used (e.g. H_100_, Lorey's height, etc.).

In both the LiDAR and SAR cases allometric equations are required to determine biomass that entail a high degree of uncertainty. Allometric equations are traditionally based on the properties of individual trees, with power law relationships between DBH, stem height, or a combination of the two. Now that height is measurable over large areas, there is growing interest in the allometry at the stand or plot level, so that the allometry takes the form:
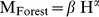
(1)where M_Forest_ is standing forest biomass, H is some average forest height, and β and α are parameters that vary as a function of species, forest type, etc. An average height is used because (or since) maximum height is not a good indicator of forest volume. The focus of this paper is to evaluate the following alternative height descriptions as indicators of standing forest biomass: mean height, H_100_ and Lorey's height, and to consider how each relationship to biomass varies with respect to population, species, resource, and area variations.

To achieve this, the forest growth model SERA (Spatially Explicit Reiterative Algorithm) is used to investigate the height-volume relationships at plot scale of simulated forests [13]. This allows the evaluation of several different descriptions of height as an indicator of plot level volume. Our link to biomass depends on the assumption that wood density is relatively consistent for any given forest composition, with genus level means giving reliable approximations of species values [14], and cross species examples explored in terms of both biomass and volume units where wood density variations may impact on results.

## Methods

### 2.1 SERA

SERA ([13], available at https://github.com/seanth/SERA) models tree growth within a population through the incorporation of ensemble behavior. Due to the inherent constraints of space and light within SERA and the allowance of species variation, it is able to mimic forest dynamics resulting from competition for light and space. As an output, SERA provides information regarding canopy size and composition as well as stem information including volume, weight (based on species-specific wood density), size, and location. SERA can be programmed to model a user-defined area, as well as user-defined conditions such as light intensity and the location and number of seeds planted. In all cases the topography is flat. The model can also be set to span a user-defined number of years. SERA has accurately predicted several relationships that have been identified within an empirically modeled *Abies Alba* population [15]. Of these relationships the two of particular importance, and the reason for this model's significance here, are the relationships between mass/volume and height, and of height to diameter. The model is used here to investigate the variations in these relationships when forest community conditions are altered in terms of number density, resource availability, and species variation.

The underlying calculations used by SERA to determine the growth of individual trees within the simulated space are made using five relationships: M_S_ 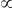
G_N_, D_S_ 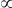
M_S_, M_L_ 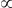
M_S_, H_S_ 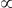
D_S_, and G_N_ 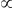
A_L_M_L_, where M_S_ is the total above ground wood mass, G_N_ is new total growth, D_S_ is the diameter of the trunk at breast height (DBH), M_L_ is the total canopy mass, H_S_ is the total height of the tree, and A_L_ is the projected area of the canopy. Of particular importance to this study are the specific equations which SERA uses to calculate tree height:
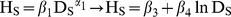
(2)where 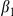, 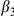 and 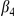 are species-specific constants (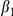 being a function of bulk stem density (sensu [16], [17]) and 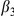 being maximum average tree height), and 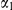 is a scaling exponent [13].

The transition from geometric self-similarity (the left hand side of the Equation (Eq. 2a)) to geometric nonsimilarity (the right side of the equation (Eq. 2b)) is determined by the growth of the individual and is not controlled by an explicit user-defined setting. When Equation 2b is greater than or equal to Equation 2a SERA makes an irrevocable swap from Equation 2a to determine height and begins using Equation 2b. The practical result of this is that young trees exhibit growth in height relative to diameter that fits the classic allometric relationship. However, as the tree reaches maturity its growth in height relative to diameter slows. In terms of tree growth in simulations, this relationship means that shaded trees will use Equation 2a for a longer period than individuals which are not shaded, since shading by neighbors reduces growth.

Within SERA each plant is intentionally simplified to consist of a single photosynthetic surface elevated by a single stem, but in this work the canopy is only used to determine ensemble growth while the stem is used to determine volume/biomass. SERA has the ability to predict the fate of a species under varying degrees of spatial and temporal heterogeneity, primarily through space and light variations.

#### 2.1.1 Allometric Comparison

SERA allometry was derived from the Cannell dataset [19] which allowed an analysis of a large range of primary literature published prior to 1982. As a comparison in Table 1 an independent study of 279 allometric studies of both angiosperm and conifer species is found in [18] where a comparative study of three methods for simplifying allometric equations of aboveground biomass (AGB) estimation are reported. The study was based on a metadata set derived from published AGB allometry conducted for different worldwide species. The statistics of variation in the scaling exponent 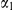 were shown to have a mean value of 2.37 with a standard deviation of 0.27 and variance 4.71. The observed SERA values for Abies Alba and generalized species are found within a single standard deviation of this value. The variation in exponents in Table 1, even for single species, highlights the variability of within-species allometry at different locations. See [13] for additional allometric values used.

**Table 1:** Reported scaling exponents α_1_ for H-D relationship taken from referenced literature.

[Ref] Study	Species	α_1_	Study	Species	α_1_	Study	Species	α_1_	r^2^
[13] Hammond et al.	SERA Silver Fir (*Abies Alba*)	2.54 (2.54)	[55] Menguzzatto et al.	Eucalyptus	2.26	[56] Woods et al.	Spruce	2.36	0.98
[13] Hammond et al.	SERA Generalized Conifer	2.48 (2.44)	[57] Baldini et al.	Maritime Pine	2.04	[56] Woods et al.	Aspen	2.42	0.99
[13] Hammond et al.	SERA Generalized Angiosperm	2.63 (2.66)	[58] Woodwell et al.	Pitch Pine	2.34	[59] Santa Regina et al.	Scots Pine	2.03	0.99
[60] Makela et al.	Scots Pine	2.69	[58] Woodwell et al.	Scarlet Oak	2.19	[61] Regina et al.	Beech	2.43	1.00
[62] Vanninen et al.	Scots Pine	2.70	[63] Cantiana	Silver Fir	2.27	[64] Jokela et al.	Paper Birch	2.36	0.97
[65] Parresol	Willow Oak	2.17	[58] Woodwell et al.	White Oak	2.17	[55] Menguzzatto et al.	Douglas Fir	2.30	0.95
[66] Taras et al.	Sand Pine	2.38	[67] Tahvanainen	Willow	2.54	Cerny et al. in [68] Schulze	Norway Spruce	2.19	0.99
[55] Menguzaatto et al.	Monterey Pine	2.29	Zianis and Mencuccini -unpublished	Beech	2.31	[69] Ketterings et al.	Tropical	2.59	0.95

Observed values for SERA species are included with the predicted values given in parentheses. Adapted from [18].

### 2.2. Height Classifications and Remote Sensing

While the height of a tree can be defined in one way – i.e. the distance of the maximum point vertically from the ground surface (although other height measures may be defined for specific purposes) – the average height of a community of trees can be described in several ways. The *maximum canopy height*, H_max_, represents the height of the tallest tree; the *mean height*, H_mean_ represents the arithmetic mean of the summed trees; H_100_ represents the mean height of the 100 trees with the largest DBH within one hectare; *Lorey's height*, H_Lorey_, refers to the mean height of the trees but with each weighted by their basal area. With the ability to quantify community height in several ways it is important to consider how heights obtained from SAR, LiDAR and traditional Optical remote sensing compare to these various height descriptions. Mean canopy height is extremely difficult to measure in the field due to the need to account for every single tree (additionally, due to its unweighted nature it is easily biased by especially large or small individuals). Mean height can be simply the arithmetic mean but can also be sample based if all trees are not measured (typical of larger stands). H_100_ remains less complicated due to the requirement to identify and measure only 100 trees per ha. H_max_ is the simplest measurement due to the need to identify and measure only the single largest tree. H_100_ [20] and H_max_ [21] are expected to resemble one another very closely particularly for mature, resource-balanced, forests. H_Lorey_ is given in equation (3) [22].
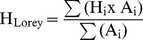
(3)H_i_ and A_i_ represent the individual characteristics of each tree within the sample area in terms of height and area respectively. Acquiring such field measurements can be difficult due to the need to measure all trees, but since the impact on the value diminishes with stem size, the omission of smaller trees is less of a problem. If we assume that crown size is approximately correlated with basal area, then H_Lorey_ is also an area-weighted mean, making it very appropriate for remote sensing, given that any pixel-based height determination will be influenced most by the larger trees. H_Lorey_ puts greater emphasis on the larger trees in a similar way to what we might expect from area-based height estimates from SAR or a large-footprint LiDAR.

### 2.3 SAR Inferred Forest Height

Synthetic Aperture Radar (SAR) is a coherent sidelooking RADAR remote sensing tool which employs microwaves (wavelengths 0.3–3 m) to generate high resolution imagery. As an active instrument it gains information from measuring the intensity of the backscattered radiation as well as through phase differences between signals of different polarization, or signals separated by location or time (referred to as SAR interferometry). Forest height retrieval using SAR interferometry has been employed as a technique for more than a decade and may be achieved using different approaches: single pass interferometry [23], [24], [25], [26]; repeat pass interferometry [27], [28], [29], [30], [28], [31]; single baseline polarimetric interferometry [32], [33], [34], [35], [36], [37]; multi-baseline interferometry [33], [38]; and multi-baseline polarimetric interferometry [39].

SAR interferometry measures a height corresponding to the “scattering phase centre”, a weighted mean of all the contributing backscatter throughout the depth of the canopy. For dense forests and short wavelengths the scattering phase centre will be close to the canopy top, while for sparse forests with gaps or at longer wavelengths, it will be closer to the forest floor [40]. Canopy height is retrieved using physical or empirical models, and the accuracy of such retrievals is restricted by the interferometric coherence [37].

### 2.4 LiDAR Inferred Forest Height

Light Detection and Ranging (LiDAR) is an active range-measuring technique similar to SAR but operating in the visible or near infrared region of the EM spectrum. Airborne LiDAR is commonly used for remotely mapping forests remote and can be either large or small footprint depending on the trade off of spatial coverage vs resolution. The shorter wavelengths and higher frequencies used in LiDAR enable it to produce high resolution images (<1 m) and highly accurate georeferenced elevation data. In order for LiDAR to calculate forest height, identification of the ground is also required. A canopy surface model is generally derived from the distribution of the first returns while the terrain model is generated through the filtering of the last returns to isolate ground reflections. For full waveform LiDAR, canopy height is calculated through analysis of the full vertical profile [41]. Large footprint systems are most effective when the canopy profile metrics are to be derived while the use of small footprint systems are applicable for more small scale surveys related to forest management, as crown diameter can be estimated and species identified.

Comparative results of LiDAR against InSAR canopy height estimation have been published in work such as [42] and [43].

## Results

### 3.1 Forest Height Analysis

SERA was used to produce forest stands of both angiosperm and gymnosperm communities. With *Abies Alba*, European Silver Fir, being the most thoroughly researched forest structure input into the SERA model it is important that this species features heavily. *Cryptomeria*, Cedar, is also included as a specific species while generic representations of angiosperms and gymnosperms are also included. For each forest identity, planting densities are varied to cover the possibilities of 1, 100, 1000, 10000 and 25000 initial seedlings per hectare (per the *Abies Alba* source plot—see [13]). The level of influence of number density, volume, basal area, height, space and light intensity (resource availability) on the forest dynamics was extensively investigated in order to distinguish what various forest height measures reveal about forest volume.

### 3.2 The Influence of Number Density

When the number density is high, competition for light means that individual trees will grow with tall and thin stems with less emphasis on mechanical stability due to the sheltering effects of neighbors. Canopy components would be solely located in the upper realms of the stem due to light competition. Conversely, under low number density trees grow with reproductive capability and mechanical stability in mind. The result is that different distributions of height and DBH result.

**Figure 1:**
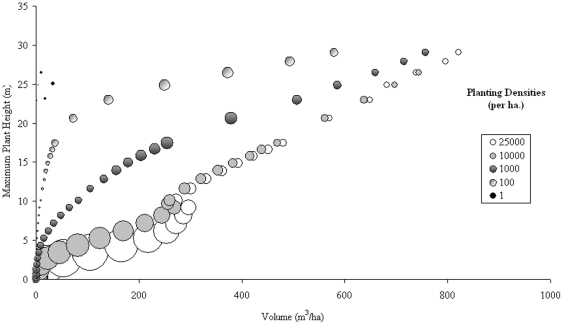
*Abies Alba* H_max_ over 100 years against stand volume. Larger circles represent larger number densities varying from the original planting density values denoted in legend as a result of new growth and mortality.

The same trend is apparent between H_max_ and Stand Age for all planting densities simulated by SERA but when H_max_ values are plotted against stem volume (Figure 1), correlation is most evident between the high density cases of 10000 and 25000 stems ha^−1^. The problems related to biomass estimation using height-based allometry are immediately apparent. For example, a SERA generated forest with an H_max_ of 25 m could be contained within a forest volume range approximately from 50–700 m^3^ ha^−1^, see Figure 1. Although the allometry suggests that the H_max_ of a plant will relate favourably to the volume it appears through SERA predictions that such a relationship is less consistent for the community scenario.

For each species the number density clearly affects the relationship between height and volume, and the H_max_ values are achieved at lower volumes when the initial planting density is less. This demonstrates that forest H_max_ to volume is a relationship which relies on the total basal area or planting density of the stand to define it. The maximum H_max_ of the forest is uniform across all planting densities. This does not signify a strong relationship but rather tells us that forest configurations eventually converge to replicate one another in a space filling and constant resource environment. If this is a common case then it is possible that the knowledge of number density at this stage of growth may be used to infer forest volume.

**Figure 2:**
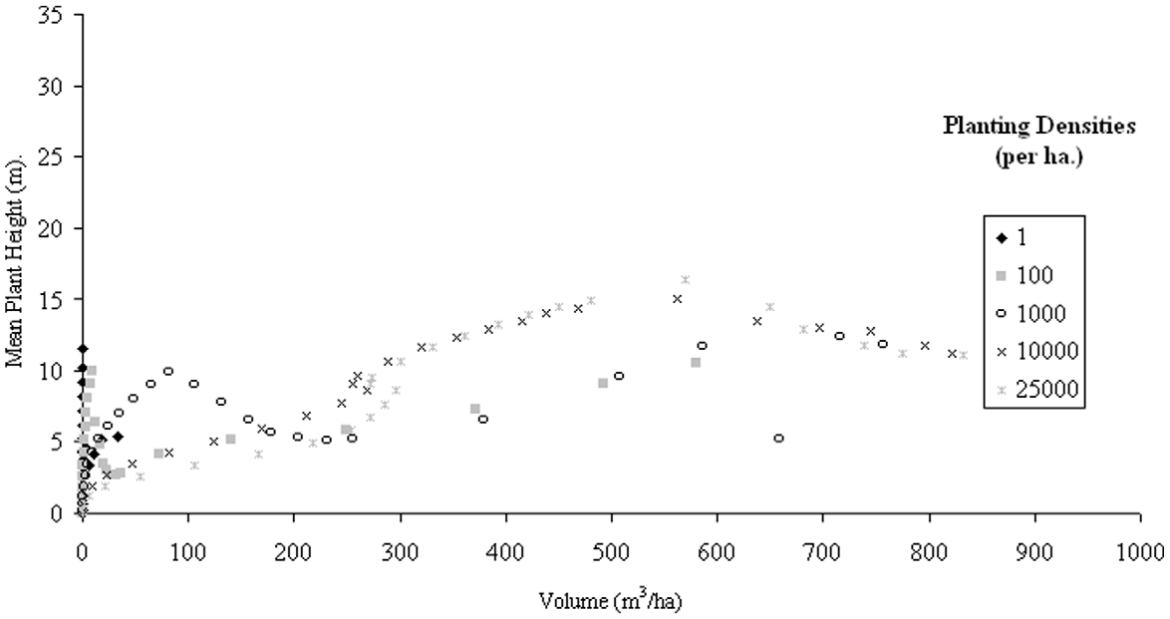
*Abies Alba* H_mean_ against stand volume over a period of 100 years.

**Figure 3:**
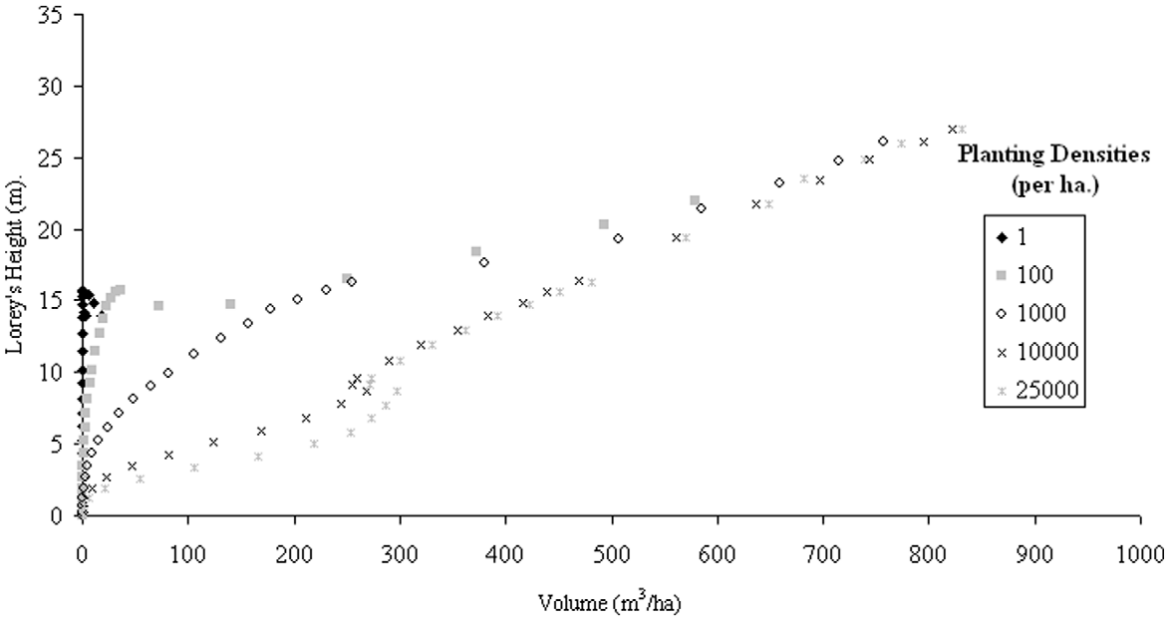
*Abies Alba* H_Lorey_ against stand volume over a period of 100 years.

With such variation in H_max_ for particular volumes, an assessment of the number density relationship to H_100_, H_mean_, and H_Lorey_ is explored, with immediate results showing a better relationship between H_mean_ and volume under number density variations (Figure 2), with the relationship of volume to H_Lorey_ (Figure 3) improving slightly on the relationship exhibited by H_max_. H_100_ is not shown here as it largely follows the trends of H_max_, particularly with large planting densities with these heights best suited to establishing forest age rather than volume.

### 3.3 The Influence of Species Variation

**Figure 4:**
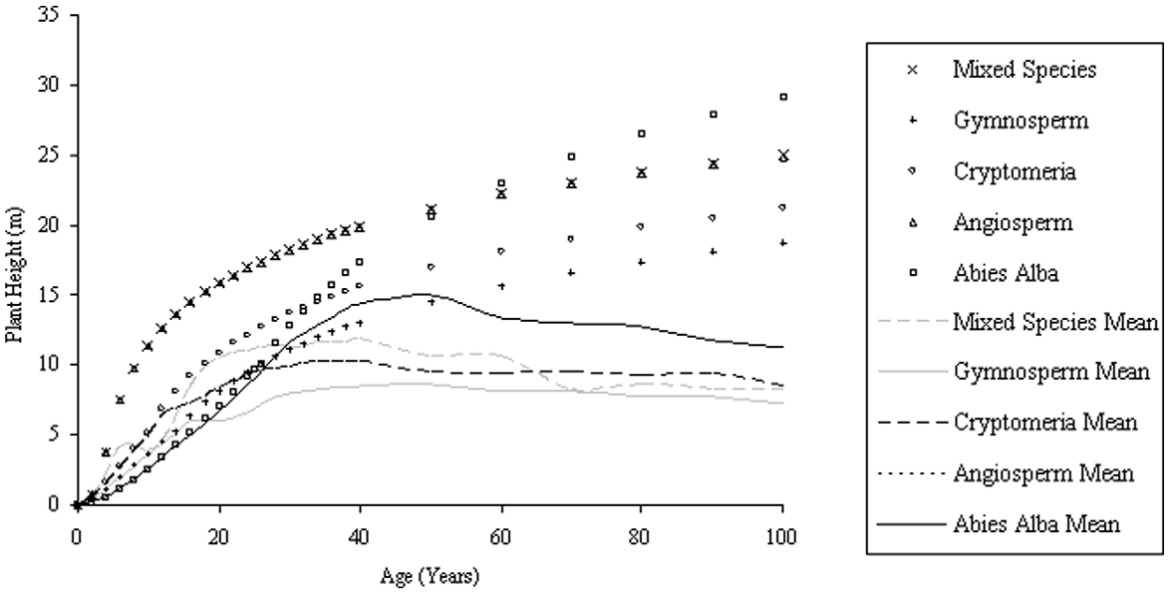
H_max_ within forests of initial planting density of 10000 stems ha.^−1^ plotted alongside H_mean_ values where indicated in the legend.

**Figure 5:**
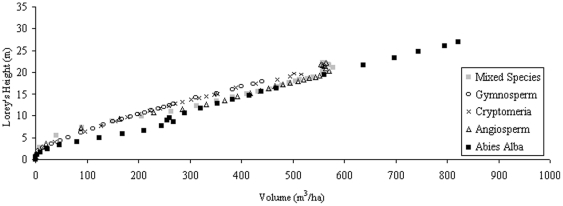
H_Lorey_ against forest volume for planting densities of 10,000 ha.^−1^.

**Figure 6:**
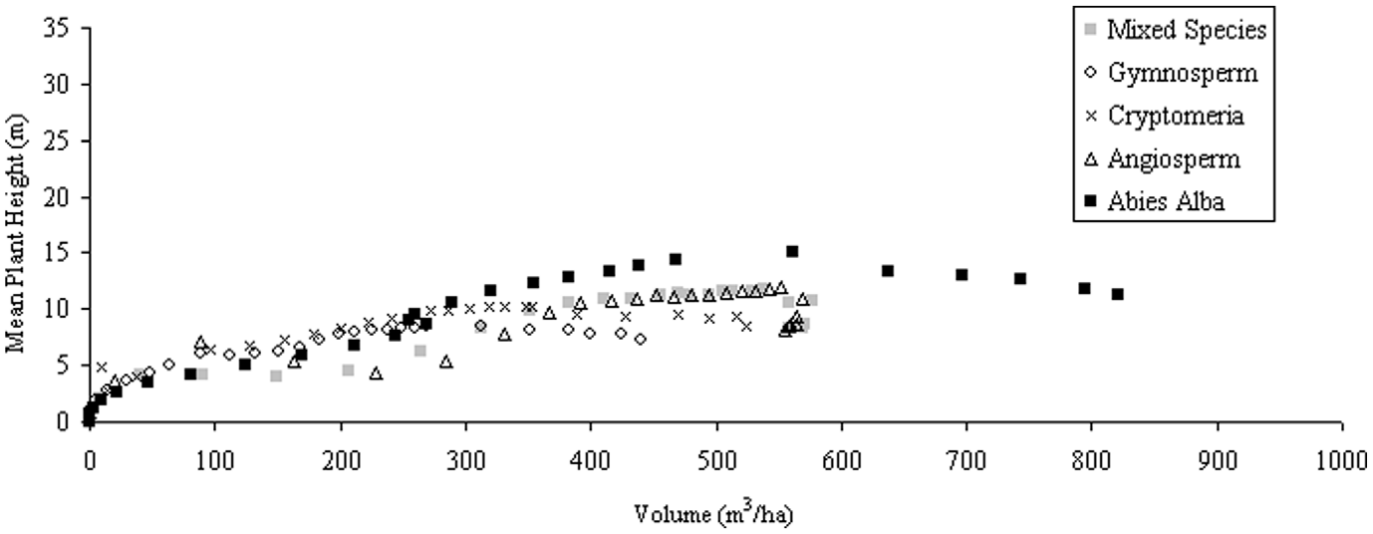
H_mean_ against forest volume for planting densities of 10000 ha^−1^.

Figure 4 shows the variations that exist with age for H_max_ and H_mean_ as a consequence of species variation at a single planting density. Each data set exhibits behavior to suggest the existence of a species optimum H_mean_ over the time period in question. When these height data are plotted against volume it appears that H_max_ is a good indicator of forest volume at volumes above 300 m^3^ ha^−1^ across all species when planting density is constant, with similar conclusions for H_100_ and H_Lorey_ (Figure 5). H_mean_ (Figure 6) as a comparison produces trends that indicate its potential as a useful parameter for indicating forest volume regardless of species (up to some maximum).

Species has a relatively small effect on the relationship between H_mean_ and forest volume in comparison to planting density variation. Although primarily H_mean_ and then H_Lorey_ appear to be the most consistent height classifications for volume estimation on an interspecies level, the rate of change is so small that it does not make it a useful property to focus on when considering remote sensing.

The impact of planting density raises the question of whether an ancillary measurement of number density could be sufficient for determining volume across species using a remotely retrieved height and quantifying the potential errors in estimation using plots such as those of Figures 5 and 6. The data in Figure 1, showing the effects of planting density on the relationship suggest this could be possible in mature forests where number densities are predicted by SERA to converge.

**Figure 7:**
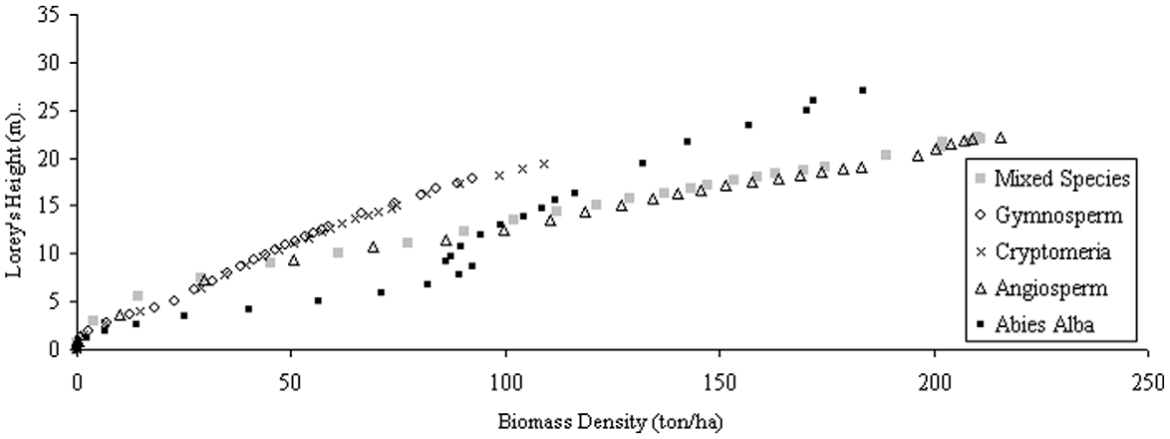
H_Lorey_ against stem biomass density for planting densities of 10,000 ha^−1^.

Also important when looking at AGB retrieval across species is the impact of wood density variations from species to species. SERA has the ability to predict mass based on field calculated wood density values for each species and although small differences are exhibited the general trends remain the same with H_mean_ continuing to produce the greatest correlation with biomass density across different species. Comparison of H_Lorey_ to both volume and biomass density highlights how difficult it is to correlate across species (Figure 7) with H_Lorey_ only improving slightly on the correlation observed for H_100_ and H_max_.

**Figure 8:**
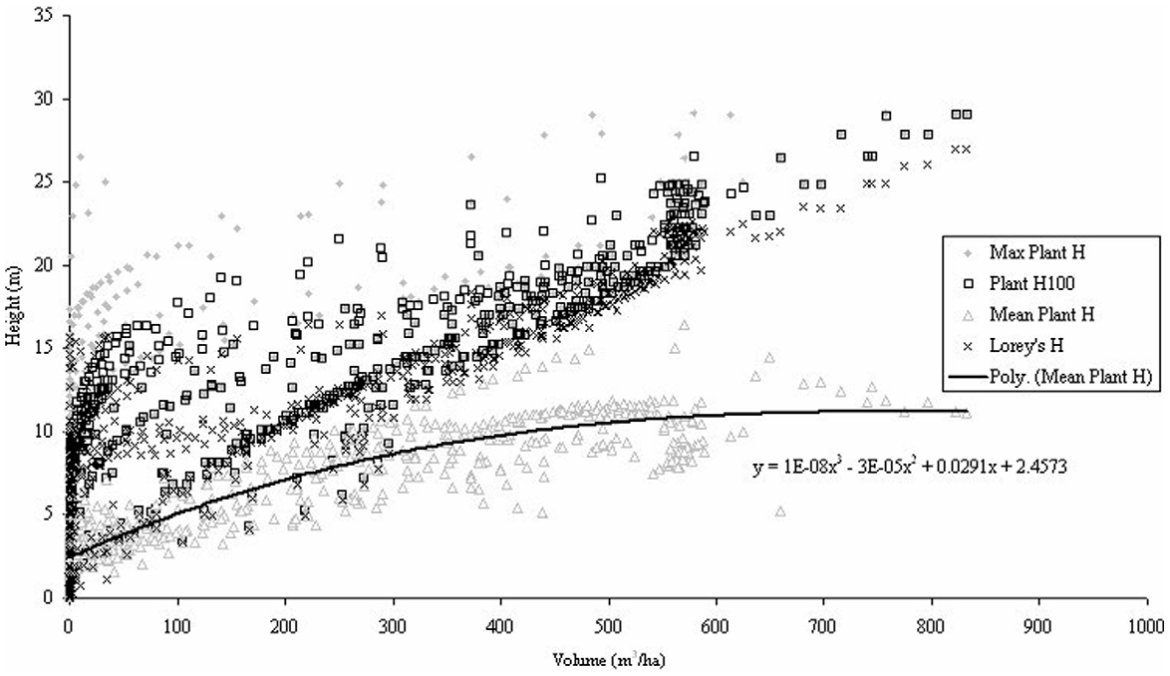
Height data for all featured forest configurations under the same environmental conditions of light intensity and space.

Tables 2 and 3 contain the r^2^ (of best fitting cubic polynomial) data relating each height classification regardless of species (Table 2) and for the best fits associated with each height classification for each individual species dataset (Table 3). The “All Data” section in Table 2 therefore provides information for the fit of all datasets combined in this study for each height classification. Table 3 represents the identification and use of individual species-specific equations for each height classification. Figure 8 shows the spread of the height data with respect to the volumes predicted by SERA for all data sets. Note the best fit equation for H_mean_ which represents the particular Mean Plant H “All Data” equation used in Table 2 in which it is applied to the collective dataset and then to the individual species in turn. The different values of r^2^ shown in the two tables highlights how knowledge of species does not necessarily lead to a better relationship between height and volume but emphasizes the influence of number density variations.

**Table 2:** r^2^ values comparing best fitting curve of height classes to forest volume generated from the combination of all forest datasets of default resources with individual forest composition examples.

	Max Plant H	Max Stem H	H_100_ Plant	H_100_ Stem	Mean Plant H	Mean Stem H	Lorey's Height
**All Data**	0.51	0.51	0.73	0.73	0.75	0.76	0.71
***Abies Alba***	0.47	0.47	0.75	0.75	0.64	0.66	0.62
**Cryptomeria**	0.56	0.56	0.78	0.78	0.86	0.86	0.75
**Generic Angiosperm**	0.60	0.60	0.75	0.75	0.82	0.82	0.79
**Generic Gymnosperm**	0.55	0.55	0.72	0.72	0.78	0.78	0.73
**Mixed Species**	0.65	0.65	0.81	0.81	0.88	0.89	0.85

Equation best representing the combination of all datasets is referred to as the “all data equation”.

**Table 3:** r^2^ values comparing best fitting curve of height classes to forest volume associated with each individual forest composition data set with default resources.

	Max Plant H	Max Stem H	H_100_Plant	H_100_ Stem	Mean Plant H	Mean Stem H	Lorey's Height
***Abies Alba***	0.45	0.45	0.70	0.70	0.64	0.66	0.50
**Cryptomeria**	0.51	0.51	0.71	0.71	0.90	0.64	0.73
**Generic Angiosperm**	0.49	0.49	0.70	0.70	0.82	0.82	0.82
**Generic Gymnosperm**	0.50	0.50	0.70	0.70	0.81	0.81	0.73
**Mixed Species**	0.55	0.55	0.79	0.79	0.88	0.89	0.82

Predicted values used for comparison were generated using best fitting curve from SERA generated data for each individual dataset of each height classification.

### 3.4 The Influence of Environmental Conditions

When discussing the influence of environmental conditions on forest height dynamics, the factors that have the most significant effect on the growth of the forest are related to the life cycle. Forest growth requires light and carbon dioxide, water, space, and nutrient availability. With SERA, the dynamics of the forest in relation to light intensity can be manipulated as well as the ability to constrain the area in which the forest can grow.

#### 3.4.1 Light Intensity

Forests experience different light intensities depending on their latitudinal location due to the angle of illumination, increased atmospheric path length and larger seasonality. This section considers the consequences of light intensity reduction predicted by SERA on height to volume relationships.

For the *Abies Alba* datasets the variations resulting from light intensity fluctuations appear to apply across all planting densities. The general trend over a 100 year period is for forests exposed to lower light intensities to grow slower in time, but on average at a faster rate of height per unit volume due to the forest accumulating less carbon over time for height gain as a result of reduced resources. Ultimately over the 100 year period average heights and total volume accumulated are less for the low light intensity. The variations are a result of increased self-thinning per unit volume within the forest to enable each surviving tree to capture the same level of light required for growth. The 100% light intensity stand will therefore allow more stems to grow to their maximum potential resulting in higher trees and higher volumes in part due to a higher and efficient rate of thinning per year.

Due to the variations in forest structure caused by light variations, the relationship of H_max_ to forest volume is not the same across all light intensities and is affected proportionally by the amount of light intensity reduction. A greater rate of change of H_max_ with volume is displayed for lower intensities. Similar findings are evident for the H_mean_ of the forest but with the surprising aspect being that forests subjected to lower intensity light can produce the maximum values of forest H_mean_, predicted for *Abies Alba*, at low planting densities. This trend suggests that there are fewer smaller trees at particular times due to the low light intensity therefore the H_mean_ would be biased to the size of the more abundant older and larger trees. Although self-thinning rates are altered by the variation in light, the allometry of trees is not predicted by SERA to vary.

**Figure 9:**
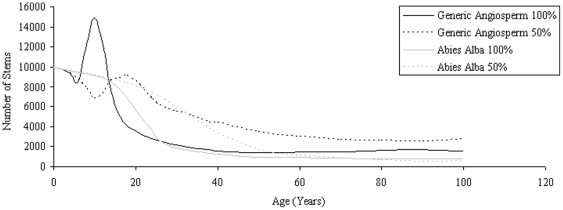
Thinning with respect to age for *Abies Alba* and Generic Angiosperm. Planting densities of 10000 ha^−1^. Light intensity variations shown in key.

For the generic angiosperm cases the rate of thinning is different to that seen for *Abies Alba* (Figure 9). The most significant difference being that, following early mortality, there is a greater surge in new growth seen for Angiosperms. The populations under light constraints produce reduced levels of this regrowth at later stages in accordance with light reduction. For angiosperms under light intensity restrictions it is difficult to relate the H_mean_ of the forest to the volume contained within with H_mean_ almost constant as forest volume increases.

The Angiosperm communities also show evidence of an optimum volume governed by light intensity, with the optimum value reducing as the available light resource is diminished. H_max_ for a particular volume still remains higher in the presence of greater light intensity, for all species. The H_mean_ is also much more closely related to volume regardless of light intensity but again, the shallow gradient raises issues about its usefulness for remote sensing purposes. This in practice could signify a lack of a durable relationship between H_max_, H_100_ or H_mean_ with volume under the constraints of light limitation, but the relationship with H_Lorey_ does not suffer in the same way, with data suggesting a more general level of increase in height observed for all increases in stand volume.

**Figure 10:**
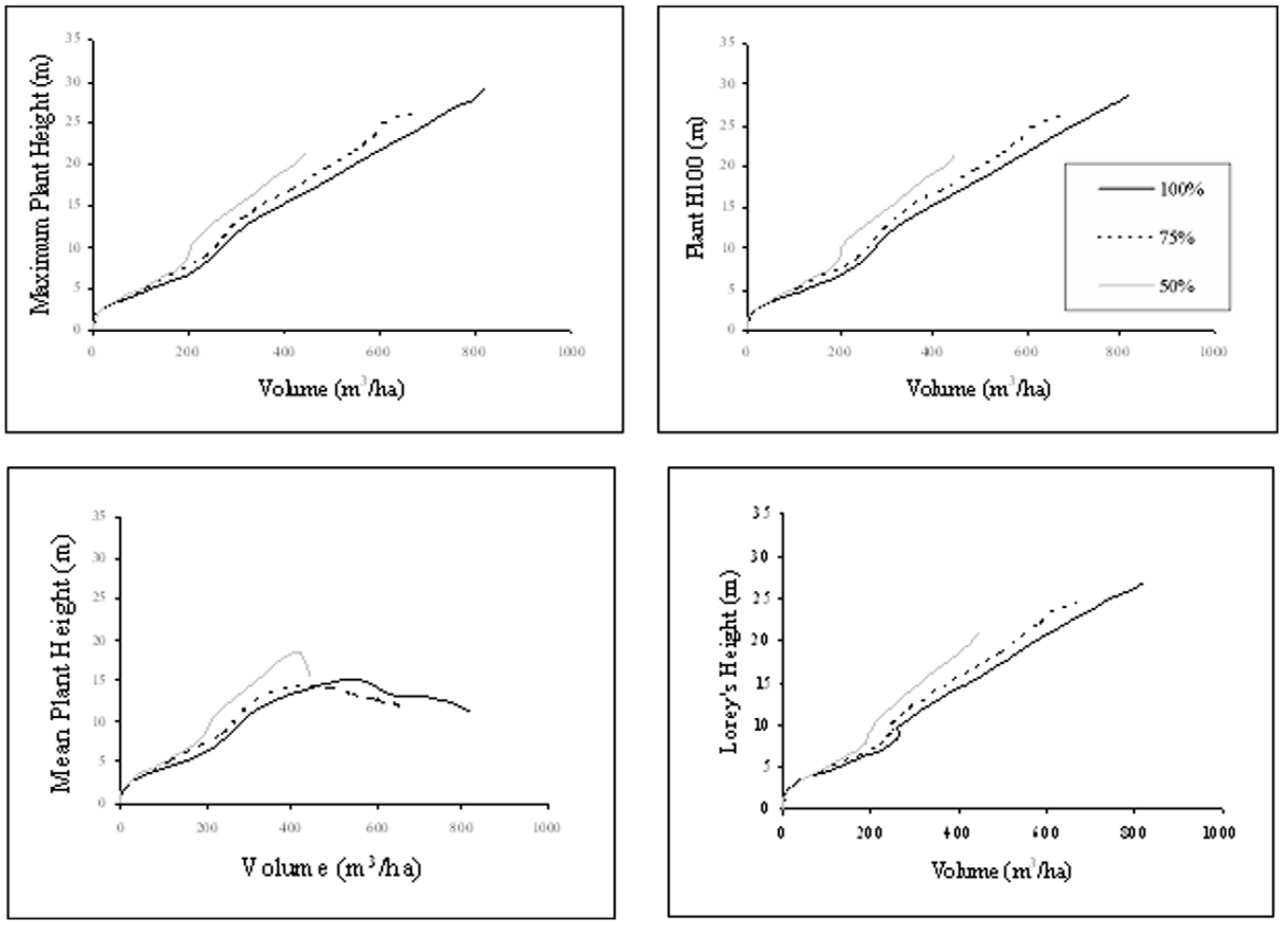
*Abies Alba* stands of planting density 10000 ha^−1^ exposed to variations in light intensity (100%, 75%, 50%). Data shown clockwise for H_max_, H_100_, H_Lorey_ and H_mean_.

Figure 10 shows how light variations are evident in the relationship of height to volume for each height classification. For each species, when light intensity is reduced, the number of stems making up a particular volume reduces also in proportion. Trees under light restrictions are bigger for any particular forest volume and typically older than those subject to more light intensity for the same stand volume. Additionally for the Abies Alba case SERA predicts that after 100 years the stand with the least light will consist of a similar number of trees to its more intense counterpart (Figure 9) but with lower collective volume and H_mean_ as growth has been stunted. This does not contradict the findings of Figure 10 as the mortality rate and subsequent regrowth is crucial in determining a forest's condition at a defined moment in time. Higher forest volumes are assumed to produce higher average heights at any particular time therefore the maximum volume over the 100 year period is significantly lower for the stands exposed to reduced light intensity as seen in Figure 10. All volumes show a lower basal area for lower light intensity, highlighting effects of limiting resources.

## Discussion

### 5.1 The Relationship Between Forest Height and Volume

All species and planting density data exposed to 100% light intensity over a 1 ha area are plotted in Figure 8 in the form of H_max_, H_mean_, H_100_, and H_Lorey_. The variations due to planting densities can be clearly seen for the data of H_100_ and H_max_ in which both show similar trends, albeit at different height levels. H_Lorey_ is also affected but shows a tighter relationship with volume. On the other hand the forest H_mean_ shows a consistent correlation with the volume of the forest as highlighted by the line of best fit; a cubic polynomial producing an r^2^ value of 0.75 between predicted and actual H_mean_, also shown in Table 2.

Regardless of species, planting density or basal area, the relationship between H_mean_ and volume remains more consistent than the other height classes investigated over all species, collectively or individually. Correlations are further improved on removal of stems shorter than 2 m high, but in doing so, the accuracy of the macroecological forest description is reduced.

The relationship between H_max_ and volume produces an r^2^ value of only 0.51 for the combination of all datasets using the “all data equation” and thus appears clear that this parameter is not a good indicator of forest volume. Angiosperm and Gymnosperm communities are represented well by their relationship of forest H_mean_ to forest volume, but poorly represented by H_max_. On a singular species level the correlation of H_Lorey_ is deemed high with the exception of the *Abies Alba* data set, for which H_100_ provides a better correlation, and suggests that H_Lorey_ is the more applicable of the two measurements for use across species. For the generic relationship between height and volume using all species data the correlation of H_100_ with volume is slightly higher when referred to all datasets but when applied to three of the five species compositions it is H_Lorey_ that is better correlated. Of 11 scenarios displayed in Tables 2 and 3, 7 cases show H_Lorey_ with better correlation than H_100_.

**Figure 11:**
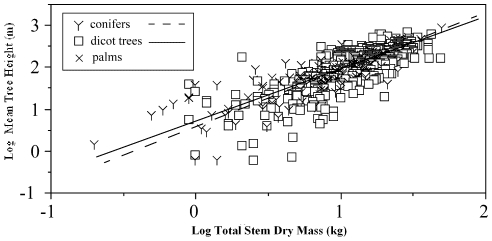
Bivariate log-log plot of tree H_mean_ against total stem, mass. Data shown for conifers, angiosperm trees and palms documented in Cannell (1982) and Luo (1996).

The results from the SERA simulations are consistent with empirical data, particularly those that indicate that tree H_mean_ is a reliable predictor of standing above-ground dry mass across forests world-wide (Figure 11). For comparison, data for H_mean_, total stem dry mass per hectare (trunk, branches, and bark; *M_s_*), total above-ground (stem and leaf) dry mass per hectare (*M_ag_*), and total basal stem area per hectare (*A_tb_*) across conifer and angiosperm dominated forested communities were collected from the Cannell world-wide compendium for forest productivity [19] and from the Luo data set for the main forest types of China [44] (see [45]). Ordinary least squares regression (OLS) protocols were used (rather than Model Type II regression protocols) because the objective was to assess the extent to which H_mean_ served as a predictor of the other variables of interest. As a result OLS regression shows that variation in H_mean_ accounts for between 42% and 74% of the variation observed for *M_s_*, *M_ag_*, and *A_tb_* across angiosperm and conifer dominated forested communities and that H_mean_ is a more effective predictor for conifer as opposed to angiosperm forests (Table 4). For the pooled data (i.e., angiosperm and conifer forests collectively), H_mean_ accounts for 67%, 66%, and 45% of *M_s_*, *M_ag_*, and *A_tb_*, respectively (see [45]). OLS regression of the data after sorting into different latitudinal bins did not alter the aforementioned trends. Accordingly, tree H_mean_ is a reasonably reliable predictor of total standing stem dry mass and therefore volume as shown in these quoted empirical studies and predicted by SERA. See Table 4.

**Table 4:** Summary of ordinary least squares regression of log_10_-transformed empirical data for H_mean_, total stem dry mass per hectare (*M_s_*), total above-ground dry mass per hectare (*M_ag_*), and total stem basal area per hectare (*A_tb_*) across conifer and angiosperm dominated forested communities.

Regression Variables and Taxon	n	r ^2^	F	P
**log ** ***M_s_*** ** vs. log ** ***h***				
Angiosperm trees	340	0.582	470.3	<0.0001
Conifers	322	0.738	901.5	<0.0001
**log ** ***M_ag_*** ** vs. log ** ***h***				
Angiosperm trees	331	0.584	462.6	<0.0001
Conifers	322	0.719	818.3	<0.0001
**log ** ***A_tb_*** ** vs. log ** ***h***				
Angiosperm trees	309	0.364	175.8	<0.0001
Conifers	421	0.419	302.2	<0.0001

Original units: H_mean_ in metres, *M* in tonnes; *A* in m^2^. F and P represent the F distribution and probability statistics.

When the individual forest compositions are considered separately the H_mean_ of a forest is still typically the best indicator of forest volume, with *Abies Alba* being the exception through its preferred relationship with H_100_. It may be the case that *Abies Alba* forest volume is weighted towards the small selection of larger trees. H_max_, and to a lesser extent H_100_, do not appear entirely representative descriptors of the community with these height descriptions representing only the most dominant individuals which SERA predicts do not define the volume status of the whole community. H_Lorey_ (in a similar fashion to H_100_ and H_max_) is biased towards the larger trees but given that it has better correlation than H_100_ or H_max_, yet poorer correlation compared to H_mean_, its relative success is probably due to the fact that it accounts for all trees. In the absence of remote sensing techniques sensitive to all tree sizes within a forest, in a similar manner to H_mean_ measurements, an appropriate and applicable alternative to H_mean_ must be considered. H_Lorey_ [22] proves slightly more accurate and appropriate for use in remote sensing than its closest competitor H_100_.

### 5.2 Regarding Resource Constraints

The *Abies Alba* data is used as a direct comparison of the full 1 ha, 100% light intensity area with the varying environmental configurations as shown in Figure 11.

By interpolating the data to allow a percentage analysis of the correlation of height values with volume at increments of 2 m^3^, the variation between 100% and 50% light intensity produces larger variations when H_mean_ is considered; showing an average 34% data variation compared to 22% for H_max_ and 30% for H_100_ with H_Lorey_ showing a 25% variation. For 75% light the variations are 3%, 4%, 8%, and 4% respectively. When it comes to available area with constant planting number the results, as would be expected, vary considerably from the control situation. This is particularly true for the 0.25 ha case. Under these conditions of shrinking area it is the H_mean_ which undergoes the least mean percentage variation for both the 0.5 and the 0.25 ha. areas with 38% and 102% variations respectively with results for H_Lorey_ of 67% and 163% being very similar to those produced for H_max_ and H_100_. These variations appear very high but result from 50% and 75% reductions in available area while maintaining the number of planted stems. When these areas are analysed in terms of volume per hectare the results are much more closely linked highlighting potential problems when the ground area available for forest growth is not classified correctly.

SERA commonly displays a convergence in stem numbers for all planting densities. This is evident at a volume of 300 m^3^ ha^−1^ for *Abies Alba*. Amongst planting densities, the amount of time it takes to achieve optimal conditions varies. Such behavior indicates that the H_mean_ of the *Abies Alba* forests will be the same regardless of planting density if these heights are achieved at volumes above 300 m^3^ ha^−1^ where convergence suggests almost identical forests. In this way the forest combats the obstacles of resource and space allocation by resorting to optimum structure to guarantee maximum efficiency through mortality and regrowth. In this scenario number of stems and species would be adequate to infer forest volume. SERA predicts that if a plot can sustain a particular number of trees it will do so by using the maximum allowable basal area and will thin according to this optimum value. Forest-level allometry is effectively altered without variations on an individual level. The effect this has on forest volume and height relationships is to have a higher H_mean_ and H_max_ for any particular absolute volume of forest in a smaller area.

### 5.3 The Relevance of Lorey's Height (*H_Lorey_*)

In general H_mean_ is shown to hold the strongest relationship with tree volume regardless of species variations, number density, light intensity, and stand area while maintaining initial planting numbers. As this work was carried out with consequences for remote sensing as a primary concern, the ability of remote sensing to measure average forest height are here considered. The three principle remote sensing techniques for forestry measurements of optical, LiDAR, and RADAR systems are often assumed to be capable of deducing H_max_ of the forest or H_100_, conditions allowing, but their ability to acquire H_mean_ and to verify using ground data is much less certain.

At high frequency bands such as X and C the dominant scattering device in the forest is the canopy volume scattering [46] with the height of the effective phase scattering centre dependent on the wavelength and polarization [47]. As the wavelength is increased the dominant scattering is associated with gradually larger branches [48]. Assuming a direct relationship between H_max_ and scattering phase centre is not always appropriate, even at X-Band [11].

A scattering phase centre in RADAR interferometry is, already, an “average” height rather than a direct measure of the canopy height. In the case of mid- to long- wavelength microwave frequencies this average height will be influenced by the size of the branches and stems [49]. Such weighted forest measurements are similar to those favored by H_Lorey_ or H_100_. For this reason, it is suggested here that for use at long wave microwave frequencies (P and L Bands) H_Lorey_ be used rather than H_100_ (the designated height classification proposed for the European Space Agency BIOMASS mission, [50]). H_Lorey_ allows the average forest height to be closely linked with the larger trees, but not being overly biased by a small subset of the largest trees (as in H_100_). For mono-cultures we might expect the difference to be small, but for natural, mixed-age forests, it is likely to be more significant.

Although LiDAR does not operate over similar bandwidths to RADAR it does produce height results biased towards the tallest trees. With the high extinction rate of optical sensors through forest canopies this is expected but the reduced accuracy when surveying conifer plantations means that the height recorded by the sensor will tend to be less than H_max_ of the forest. H_Lorey_ would once again be a reasonable evaluation of the inferred height from the LiDAR measurements with allometry suggesting that taller trees will have larger basal areas.

### 5.4 An Alternative Relationship

**Figure 12:**
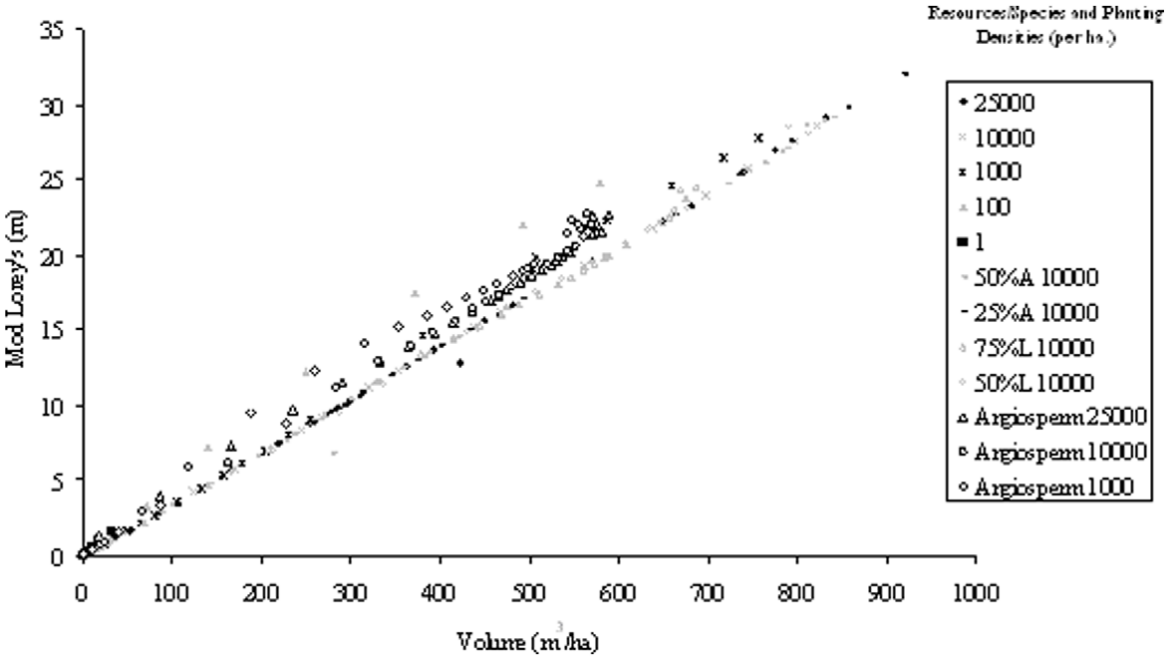
Mod Lorey height for various planting densities of *Abies Alba* and Angiosperms. Data also plotted for reduced light intensities (L) both for *Abies Alba* populations. All data plotted is taken from forests with fraction of forested area set as 1 ha. except for data represented by 50%A and 25%A. In these cases the fractional area is 0.5 and 0.25 respectively.

The problem with height as an indicator of volume is of particular significance in the cases of resource limitation and space competition. A single stem existing within a single hectare plot will provide a H_max_ that is equal to the H_mean_ which is also equal to H_Lorey_. In cases such as this the relationship of each height class with volume will be the same yet completely different from the relationships exhibited in communities of trees. With regards to interferometric SAR the height retrieved from the system will not correspond to H_max_ and therefore will not correspond to the other classes investigated in this work. In areas that meet such criteria the need to incorporate environmental conditions into a height classification are required to inform on forest volume. If the plot capability is known in terms of the total basal area per hectare it is able to support then the presence of a reduced number of stems within this area will allow the relationship between height and the volume to be refined. If for example a plot can sustain 30 m^2^ ha^−1^ of a particular species then the presence of only 3 m^2^ ha^−1^ in a scene can be deemed to be 10% of the stand capability. Within any particular collection of stands undergoing similar forest dynamics the relationship between H_max_ and volume can be constrained into a relationship following the process of equation (4) here named “Mod Lorey Height”. This process requires knowledge of the optimum basal area of the stand per ha (which may be determined from an appropriate model) as well as current basal area and H_max_ values. Difficulties arise for determining current basal area from remote sensing methods but a relationship with canopy size and cover is shown to exist for particular species [51], [52], [53], making an estimation of basal area and Mod Lorey height using remote sensing a possibility, particularly when the species is known. Area restrictions are considered by dividing the current absolute basal area by the fractional area occupied to provide the relative basal area per ha. Knowledge of species to determine the potential basal area of a stand is required in addition to the knowledge of any resource restrictions and potential for growth. This process can account for all planting densities and species for complete and partial area coverage.
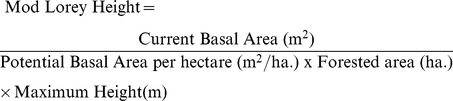
(4)Mod Lorey Height can then be plotted against volume calculated by dividing absolute volume by the fraction of the forested hectare area which throughout this study has had default of 1, resulting in a data spread as shown in Figure 12 for *Abies Alba* and Angiosperm data. This figure includes two additional datasets representing fractional areas of 0.5 and 0.25 ha. for comparison. Correlation across species, planting densities, and resource limitation show the measurement's potential.

If InSAR measurements of height were related to such a measurement as Mod Lorey, a measurement that takes into account the nature of scattering through tree size dependence and maximum height (indicating the weight and first instance of scattering), then a generic relationship with volume may be obtainable. This the subject of ongoing work by the authors.

### 5.5 Conclusions

Forest height and volume are intricately linked, but it is H_mean_ that is most indicative of forest volume; across species, planting density, and resource variation. With the success of one equation, representing all forest configurations, predicting the volume of each separate species stand based on the collective H_mean_, it is believed that this height parameter is the most accurate. The possible variations in the relationship between H_max_ and volume under the same conditions are extremely variable, even when considered within the confines of a monospecies scenario. When light is restricted it has been shown that the trees cannot grow to the same H_max_ within the time frame of the study for any particular volume, therefore SERA predicts that at the highest plant heights the relationship with volume will be unreliable. Through the same conditions, the relationship of H_mean_ remains significantly more consistent.

As a result of these findings it is important to identify when the SAR phase centre or the equivalent for LiDAR can be associated with the average height of the forest. LiDAR would be required to measure the H_max_ of each tree in order to ascertain a mean value, which is not economically or mechanically practical, and methods involving SAR are similarly complex. While H_max_ and H_100_ are reasonable predictors of volume across areas of variable resources and size, the inability of H_max_ to successfully predict volume across species boundaries, as well as amongst various planting densities, is a significant deficiency to its use in large area remote sensing. Therefore with regards to SAR remote sensing in particular, the weighting of the average height in favor of the basal area to produce H_Lorey_ allows a greater connection with the nature of microwave scattering than offered by H_100_ or H_max_. Microwave scattering is dominated by relatively larger structures according to particular ratios between the wavelength of the incident wave and the size of the object. Any scattering phase centre, if deemed to be related to average height, would be weighted towards the relatively larger structures. For LiDAR the physical connection is not as clear but appears to be valid due to its relation to the larger trees.

The variation in the correlations between the examined height classifications and their relationships with volume have shown how the way we interpret forest height can vastly influence our forest volume estimations. As the heights often used in field studies tend to be related to H_max_ (or samples of this measure) it is clear that large errors exist through association with this parameter and may be greater when used at changing locations. As the benefits of a relationship with H_100_ are less obvious and inherently less correlated with scattering physics this work recommends, in the absence of a feasible physical relationship between the remote sensing techniques used here and H_mean_, the use of H_Lorey_ as an alternative to the H_100_ measure in remote sensing studies. Even though, H_100_ still represents an improvement to using H_max_. Similarly to H_mean_, H_Lorey_ accounts for all trees, weighting the measurements towards the most dominant scatterers in a similar manner to RADAR interactions with the absence of small trees in the remotely sensed data becoming less significant. Additionally the quantitative and conceptual similarities between H_Lorey_ and “Crown-area-weighted mean height” [54] which can be defined using LiDAR measurements makes comparisons possible in the absence of basal area data.

It is important to keep in mind that this study relies heavily upon SERA, its use as a modeling tool is primarily based on its ability to predict empirically monitored behavior. The ability to vary the allometry within the model using species definition allows forests of various allometric identities to be modeled independently and collectively within SERA. In effect this study has analyzed the effects of individual allometry variations on the height-to-volume relationships of the forest through species definition. It has also, significantly, evaluated the consequences of collective forest allometry variations resulting from resource limitation and number density fluctuations to show that forest height and volume follow a complex relationship dependent on many environmental and physical factors. Self thinning rates are one such factor.
