# Correction: A Macroecological Analysis of SERA Derived Forest Heights and Implications for Forest Volume Remote Sensing

**DOI:** 10.1371/annotation/13754ad8-2763-439e-9dda-0ab882a8f203

**Published:** 2012-08-09

**Authors:** Matthew Brolly, Iain H. Woodhouse, Karl J. Niklas, Sean T. Hammond

There was an error in affiliation 4 for author Sean T. Hammond. Affiliation 4 should be: Department of Biology, University of New Mexico, Albuquerque, New Mexico, United States of America 

